# CRISPR screening identifies GNPTAB as a noncanonical STING activator driving cellular senescence

**DOI:** 10.1093/procel/pwag049

**Published:** 2026-07-27

**Authors:** Jian Yin, Yizhou Gao, Yaobin Jing, Xiaoyu Jiang, Feibo Wang, Yusheng Cai, Ming-Heng Li, Tianling Cao, Min Wang, Yifang He, Hongkai Zhao, Sheng Zhang, Shuai Ma, Si Wang, Guang-Hui Liu, Weiqi Zhang, Jing Qu

**Affiliations:** State Key Laboratory of Organ Regeneration and Reconstruction, Human Organ Physiopathology Emulation System, Institute of Zoology, Chinese Academy of Sciences, Beijing 100101, China; University of Chinese Academy of Sciences, Beijing 100049, China; State Key Laboratory of Organ Regeneration and Reconstruction, Human Organ Physiopathology Emulation System, Institute of Zoology, Chinese Academy of Sciences, Beijing 100101, China; University of Chinese Academy of Sciences, Beijing 100049, China; Key Laboratory of Reproductive Health Diseases Research and Translation of Ministry of Education, International Center for Aging and Cancer, Hainan Medical University, Haikou 571199, China; State Key Laboratory of Organ Regeneration and Reconstruction, Human Organ Physiopathology Emulation System, Institute of Zoology, Chinese Academy of Sciences, Beijing 100101, China; University of Chinese Academy of Sciences, Beijing 100049, China; State Key Laboratory of Organ Regeneration and Reconstruction, Human Organ Physiopathology Emulation System, Institute of Zoology, Chinese Academy of Sciences, Beijing 100101, China; University of Chinese Academy of Sciences, Beijing 100049, China; State Key Laboratory of Organ Regeneration and Reconstruction, Human Organ Physiopathology Emulation System, Institute of Zoology, Chinese Academy of Sciences, Beijing 100101, China; Beijing Institute for Stem Cell and Regenerative Medicine, Beijing 100101, China; State Key Laboratory of Organ Regeneration and Reconstruction, Human Organ Physiopathology Emulation System, Institute of Zoology, Chinese Academy of Sciences, Beijing 100101, China; University of Chinese Academy of Sciences, Beijing 100049, China; State Key Laboratory of Organ Regeneration and Reconstruction, Human Organ Physiopathology Emulation System, Institute of Zoology, Chinese Academy of Sciences, Beijing 100101, China; University of Chinese Academy of Sciences, Beijing 100049, China; State Key Laboratory of Organ Regeneration and Reconstruction, Human Organ Physiopathology Emulation System, Institute of Zoology, Chinese Academy of Sciences, Beijing 100101, China; Division of Life Sciences and Medicine, School of Life Sciences, University of Science and Technology of China, Hefei 230001, China; State Key Laboratory of Organ Regeneration and Reconstruction, Human Organ Physiopathology Emulation System, Institute of Zoology, Chinese Academy of Sciences, Beijing 100101, China; University of Chinese Academy of Sciences, Beijing 100049, China; State Key Laboratory of Organ Regeneration and Reconstruction, Human Organ Physiopathology Emulation System, Institute of Zoology, Chinese Academy of Sciences, Beijing 100101, China; Division of Life Sciences and Medicine, School of Life Sciences, University of Science and Technology of China, Hefei 230001, China; State Key Laboratory of Organ Regeneration and Reconstruction, Human Organ Physiopathology Emulation System, Institute of Zoology, Chinese Academy of Sciences, Beijing 100101, China; University of Chinese Academy of Sciences, Beijing 100049, China; State Key Laboratory of Organ Regeneration and Reconstruction, Human Organ Physiopathology Emulation System, Institute of Zoology, Chinese Academy of Sciences, Beijing 100101, China; University of Chinese Academy of Sciences, Beijing 100049, China; Beijing Institute for Stem Cell and Regenerative Medicine, Beijing 100101, China; Aging Biomarker Consortium (ABC), Beijing 100101, China; Aging Biomarker Consortium (ABC), Beijing 100101, China; Advanced Innovation Center for Human Brain Protection, National Clinical Research Center for Geriatric Disorders, Aging Translational Medicine Center, Beijing Municipal Geriatric Medical Research Center, Beijing Key Laboratory of Environment and Aging, Xuanwu Hospital, Capital Medical University, Beijing 100053, China; State Key Laboratory of Organ Regeneration and Reconstruction, Human Organ Physiopathology Emulation System, Institute of Zoology, Chinese Academy of Sciences, Beijing 100101, China; University of Chinese Academy of Sciences, Beijing 100049, China; Beijing Institute for Stem Cell and Regenerative Medicine, Beijing 100101, China; Aging Biomarker Consortium (ABC), Beijing 100101, China; University of Chinese Academy of Sciences, Beijing 100049, China; Aging Biomarker Consortium (ABC), Beijing 100101, China; China National Center for Bioinformation, Beijing Institute of Genomics, Chinese Academy of Sciences, Beijing 100101, China; State Key Laboratory of Organ Regeneration and Reconstruction, Human Organ Physiopathology Emulation System, Institute of Zoology, Chinese Academy of Sciences, Beijing 100101, China; University of Chinese Academy of Sciences, Beijing 100049, China; Beijing Institute for Stem Cell and Regenerative Medicine, Beijing 100101, China; Aging Biomarker Consortium (ABC), Beijing 100101, China; Beijing Institute of Heart, Lung and Blood Vessel Diseases, Beijing Anzhen Hospital, Capital Medical University, Beijing 100029, China

**Keywords:** senescence, lysosome, GNPTAB, STING, CRISPR screening

## Abstract

Cellular senescence is accompanied by profound lysosomal alterations, yet whether lysosome-associated factors actively drive aging remains unclear. Through a focused CRISPR/Cas9 screen in human mesenchymal progenitor cells (hMPCs), we identified N-acetylglucosamine-1-phosphotransferase subunits alpha and beta (GNPTAB), an enzyme responsible for lysosomal hydrolase targeting, as a positive regulator of cellular senescence. Genetic ablation of GNPTAB attenuated senescence, whereas its overexpression accelerated senescence. This pro-senescent function occurred independently of GNPTAB’s canonical enzymatic role. Instead, GNPTAB binds to the innate immune adaptor stimulator of interferon genes (STING) through an interface involving the critical residue E1119, leading to activation of STING and its downstream TANK-binding kinase 1 (TBK1), as well as pro-inflammatory gene expression. A STING-binding-deficient GNPTAB mutant (E1119A) preserved canonical lysosomal functions but failed to induce senescence, while STING depletion abolished GNPTAB-driven senescence. Together, these findings uncover a new signaling pathway wherein GNPTAB engages STING to facilitate its activation, suggesting that the GNPTAB-STING interaction interface may serve as a potential therapeutic target for ameliorating age-related cellular dysfunction.

## Introduction

The accumulation of senescent cells is a fundamental driver of organismal aging, fueling chronic inflammation, tissue dysfunction, and a spectrum of age-related diseases (Cohen et al., 2025;  Huang et al., 2022; Li et al., 2016; Li et al., 2023; López-Otín et al., 2023; Ren et al., 2017; Wu et al., 2025). Senescent cells are characterized by an irreversible cell-cycle arrest and a potent senescence-associated secretory phenotype (SASP), which together contribute to tissue remodeling and age-associated functional decline (Gladyshev et al., 2021; Herr et al., 2024; Huang et al., 2024; Li et al., 2025; López-Otín et al., 2023; Ren et al., 2023; Sun et al., 2022; Van Deursen 2014; Wu et al., 2026; Zhang et al., 2026). Among the inflammatory pathways implicated in senescence, the cyclic GMP-AMP synthase (cGAS)-stimulator of interferon genes (STING) innate immune pathway has emerged as a central and inducible effector of senescence-associated inflammation (Chiu et al., 2009; Hong et al., 2024; Tanaka and Chen, 2012; Tang et al., 2025; Wang et al., 2024; Wu et al., 2024; Zhao et al., 2025). However, the upstream mechanisms that activate or modulate STING during cellular senescence, particularly beyond canonical DNA damage-cGAS signaling, remain incompletely understood (De Cecco et al., 2019; Dou et al., 2017; Glück et al., 2017; Mackenzie et al., 2017; Thomas et al., 2017; Yan et al., 2020; Yang et al., 2017).

Lysosomal alterations are among the most recognizable features of senescent cells, as reflected by increased lysosomal mass, altered lysosomal activity, and elevated senescence-associated β-galactosidase (SA-β-Gal) activity (Ren et al., 2023; Rubinsztein et al., 2011; Settembre et al., 2013; Sun et al., 2022; Terman and Brunk, 2006; Yang et al., 2017). These observations have made SA-β-Gal, the specific lysosomal feature as a useful entry point for interrogating senescence. However, whether individual lysosome-associated factors merely reflect lysosomal remodeling or instead exert previously unrecognized signaling functions that influence senescence remains unclear. In particular, systematic genetic approaches are needed to identify such factors and distinguish canonical lysosomal roles from noncanonical pro-senescent activities (Aman et al., 2021; Faget et al., 2019; Nixon and Rubinsztein, 2024; Parenti et al., 2021; Rhinn et al. 2019; Wang et al., 2025).

To address this causality gap, we performed a focused CRISPR/Cas9 loss-of-function (LOF) screen targeting Lysosome-associated genes (LAGs) in human mesenchymal progenitor cells (hMPCs) (Li et al., 2023; Li et al., 2025; Li et al., 2024; Sun et al., 2026). This unbiased functional genomics approach identified N-acetylglucosamine-1-phosphotransferase subunits alpha and beta (GNPTAB), a core enzyme mediating mannose-6-phosphate (M6P) tagging of lysosomal hydrolases (Li et al., 2022; Marschner et al., 2011), as a top-ranked regulator of cellular senescence. We demonstrated that *GNPTAB* loss attenuated senescence while its overexpression accelerated it. Crucially, its pro-senescent function is independent of its canonical role. Instead, we uncover a noncanonical mechanism wherein GNPTAB directly binds to and activates the STING signaling pathway, thereby initiating a potent inflammatory and senescent program. Our findings define GNPTAB as a previously unrecognized, noncanonical activator of STING-dependent senescence, establishing a molecular link between lysosome-associated machinery and innate immune activation in aging.

## Results

### A focused CRISPR screen identifies GNPTAB as a senescence regulator

To causally interrogate the contribution of lysosome-associated components to cellular senescence, we conducted a CRISPR/Cas9 LOF screen in hMPCs subjected to a replicative senescence (RS) induction protocol (Fig. S1A–D). We designed a custom single guide RNA (sgRNA) library targeting 148 genes with well-defined lysosomal functions (LAGs), systematically covering lysosomal enzymes and biogenesis factors (66 genes), lysosomal enzyme-modifying proteins (13 genes), lysosomal trafficking machinery (46 genes), and lysosomal membrane proteins (23 genes) (Figs. 1A and S1E–G; Table S1). RS hMPCs were transduced with the library at low multiplicity of infection (MOI ∼0.3) to ensure single sgRNA integration, selected with puromycin, and propagated under chronic passaging pressure that enriches for cells with retarded senescence. The pooled LAGs-knockout population exhibited a proliferative advantage over cells expressing non-targeting control (NTC) sgRNAs, as demonstrated by enhanced clonal expansion, increased Ki67 positivity, and reduced SA-β-Gal activity (Fig. 1B–D). These results indicated that loss of specific lysosomal factors can delay the senescent state.

**Figure 1.**
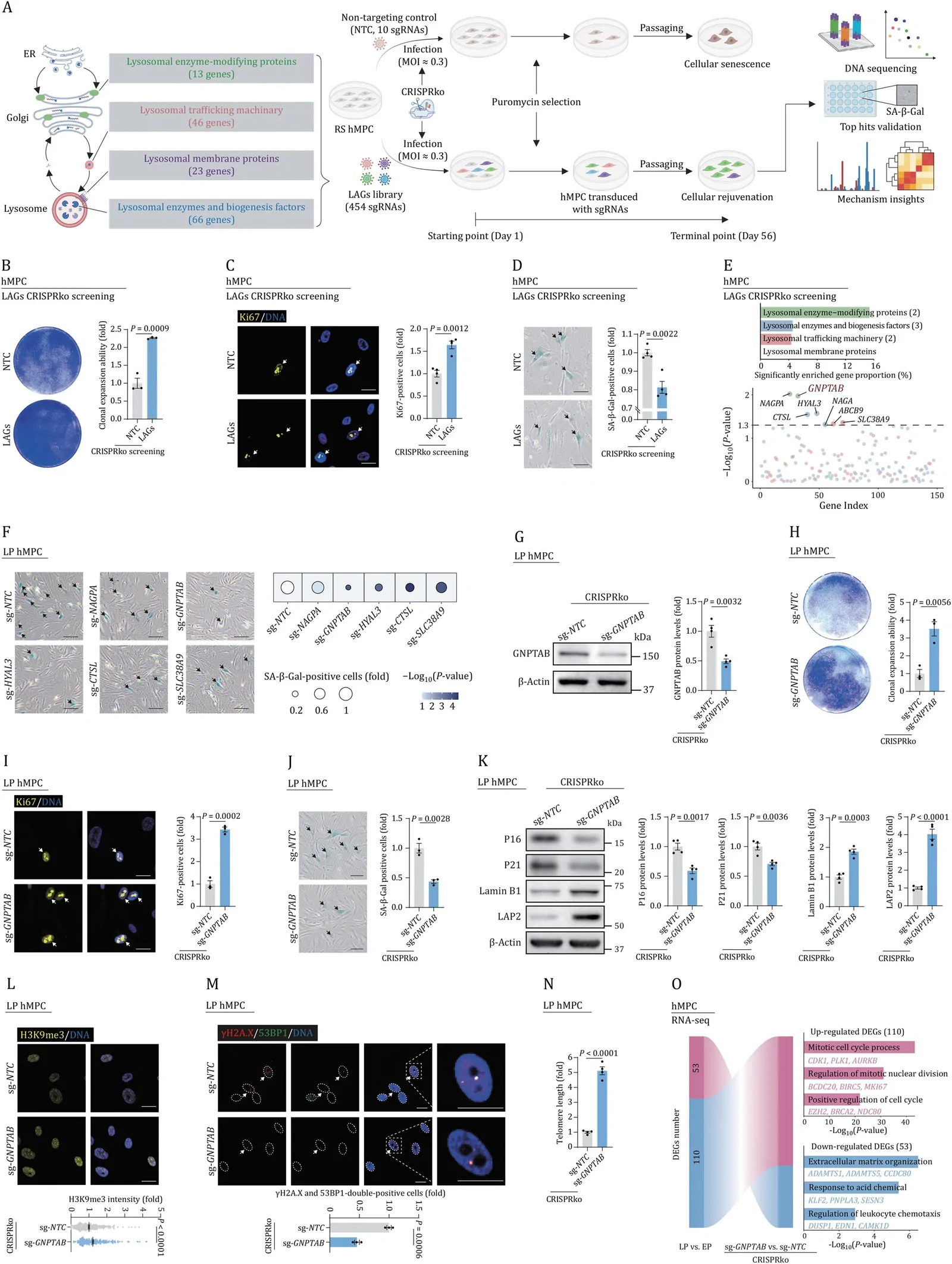
**CRISPR/Cas9 screening identifies GNPTAB as a driver of human MPC senescence**. (A) Schematic of the CRISPR/Cas9 LOF screen targeting 148 LAGs with 454 sgRNAs in RS hMPCs. (B) Clonal expansion analysis of LAGs sgRNA library-transduced cells compared with non-targeting controls (NTCs). (C) Immunofluorescence staining of Ki67 of LAGs sgRNA library-transduced cells compared with NTCs. Scale bars, 20 μm. Arrows indicate Ki67-positive cells. (D) SA-β-Gal staining of LAGs sgRNA library-transduced cells compared with NTCs. Scale bars, 100 μm. Arrows indicate SA-β-Gal-positive cells. (E) Dot plot showing gene enrichment analysis of LAGs CRISPR-mediated knockout (CRISPRko) screening in RS hMPCs. The bar chart illustrates the proportion of significantly enriched genes across the four categories of LAGs, with the number of these genes indicated in parentheses. (F) Representative images and bubble plot summarizing SA-β-Gal activity in late passage (LP) hMPCs transduced with sgRNAs targeting *NAGPA*, *GNPTAB*, *HYAL3*, *CTSL*, *SLC38A9*. Bubble size corresponds to the fold change relative to sg-*NTC*, and bubble color indicates statistical significance. Scale bars, 100 μm. Arrows indicate SA-β-Gal-positive cells. (G) Western blot analysis of GNPTAB protein level in LP hMPCs after CRISPRko of *GNPTAB*. β-Actin was used as the loading control. (H) Clonal expansion analysis of LP hMPCs after CRISPRko of *GNPTAB*. (I) Immunofluorescence staining of Ki67 of LP hMPCs after CRISPRko of *GNPTAB*. Scale bars, 20 μm. Arrows indicate Ki67-positive cells. (J) SA-β-Gal staining of LP hMPCs after CRISPRko of *GNPTAB*. Scale bars, 100 μm. Arrows indicate SA-β-Gal-positive cells. (K) Western blot analysis of P16, P21, Lamin B1 and LAP2 protein levels in LP hMPCs after CRISPRko of *GNPTAB*. β-Actin was used as the loading control. (L) Immunofluorescence analysis of H3K9me3 in LP hMPCs after CRISPRko of *GNPTAB*. Scale bars, 20 μm. (M) Immunostaining analysis of γH2A.X and 53BP1 in LP hMPCs after CRISPRko of *GNPTAB*. Scale bars, 20 μm. Arrows indicate γH2A.X and 53BP1 double-positive foci. (N) Quantitative PCR (qPCR) analysis of telomere length in LP hMPCs after CRISPRko of *GNPTAB*. The expression level was normalized to *RPLP0*. (O) The Sankey diagram showing the DEGs in *GNPTAB*-knockout hMPCs and RS hMPCs (left). The bar plot displays gene ontology (GO) terms and pathways associated with DEGs rescued by CRISPRko of *GNPTAB* (right). The upper and lower panels represent enriched pathways associated with upregulated and downregulated DEGs, respectively. Data in all figures except for (A), (E), (F), and (O) are presented as mean ± SEM. Two-tailed unpaired Student’s *t* test (B–D, F–K, M, N) and two-tailed Wilcoxon test (L) were used. *n* = 3–4 biological replicates in (B–D), (F–K), (M), (N); *n* ≥ 300 cells from three biological replicates in (L).

Deep sequencing of sgRNA representation followed by MAGeCK analysis (Li et al., 2014) identified an enrichment of sgRNAs targeting genes involved in lysosomal enzyme modification (Fig. 1E; Table S2). To validate these candidates, we individually knocked out five high-ranking genes (N-acetylglucosamine-1-phosphodiester alpha-N-acetylglucosaminidase (*NAGPA*), *GNPTAB*, Hyaluronidase 3 (*HYAL3*), Cathepsin L (*CTSL*), Solute carrier family 38 member 9 (*SLC38A9*)) in senescent hMPCs. While most hits showed some effect, knockout of *GNPTAB* produced the most robust and consistent attenuation of senescence, evidenced by the greatest reduction in SA-β-Gal positivity (Fig. 1F). Based on this pronounced and reproducible rejuvenation phenotype, *GNPTAB* was selected for in-depth mechanistic investigation.

GNPTAB is essential for catalyzing the M6P tag that directs hydrolases to lysosomes (Flint et al., 2019; Pechincha et al., 2022; Xiao et al., 2021). Confirming our screening result, lentiviral CRISPR/Cas9-mediated knockout of *GNPTAB* in RS hMPCs mitigated a broad spectrum of senescence hallmarks. This included improved proliferative capacity, as evidenced by enhanced colony formation and increased Ki67^+^ cells (Fig. 1G–I). Concurrently, we observed a reduction in canonical senescence markers, including decreased P16 and P21 protein levels. Moreover, *GNPTAB* knockout partially restored nuclear architecture, manifested as elevated levels of Lamin B1 and the heterochromatin marks trimethylation of histone H3 lysine 9 (H3K9me3), lamina-associated polypeptide 2 (LAP2), and heterochromatin protein 1 alpha (HP1α) (Figs. 1J–L and S1H–J). Furthermore, GNPTAB ablation alleviated genomic instability, manifesting as fewer DNA damage foci (phosphorylated histone H2A.X (γH2A.X)/tumor protein p53-binding protein 1 (53BP1)) and higher relative telomere signal (Fig. 1M and 1N) (Ma and Yu, 2026). Notably, these geroprotective effects occurred without compromising the multilineage differentiation potential of hMPCs (Fig. S1K–M). To globally assess the impact of GNPTAB loss at the transcriptome level, we performed RNA sequencing and integrated the data with a published transcriptomic dataset of senescent hMPCs (Liu et al., 2022). This analysis revealed that *GNPTAB* knockout partially rescued senescence-associated transcriptional alterations (Table S3). It upregulated genes linked to cell cycle and mitosis, while downregulating genes involved in pathways of chemotaxis and extracellular matrix remodeling (Figs. 1O, S1N and S10). Together, these data establish *GNPTAB* not merely as a lysosomal regulatory gene, but as a critical upstream regulator orchestrating multiple facets of cellular senescence.

### *GNPTAB* depletion mitigates senescence across diverse senescent models

To assess whether the geroprotective function of GNPTAB is generalizable beyond replicative senescence, we evaluated its role across a panel of genetically defined and stress-induced senescent models. First, we utilized Hutchinson-Gilford progeria syndrome (HGPS) hMPCs, which harbor a lamin A/C (*LMNA*) mutation leading to progerin accumulation and recapitulate accelerated aging (Wu et al., 2020). *GNPTAB* knockout in this context rescued multiple premature aging phenotypes, including restoration of proliferative capacity, reduction of SA-β-Gal activity, re-establishment of heterochromatin marks (H3K9me3, LAP2), and attenuation of telomere attrition (Fig. S2A–G).

We next asked whether GNPTAB loss could also counteract senescence driven by acute exogenous stressors. Indeed, CRISPR-mediated ablation of GNPTAB attenuated senescence induced by oncogenic H-Ras^V12^ expression, oxidative stress (hydrogen peroxide, H_2_O_2_), and genomic damage (ultraviolet irradiation, UV). In each model, GNPTAB-deficient cells maintained higher clonogenic potential, proliferative index, alongside reduced senescent load compared to controls (Figs. 2A–F and S2H–J).

**Figure 2.**
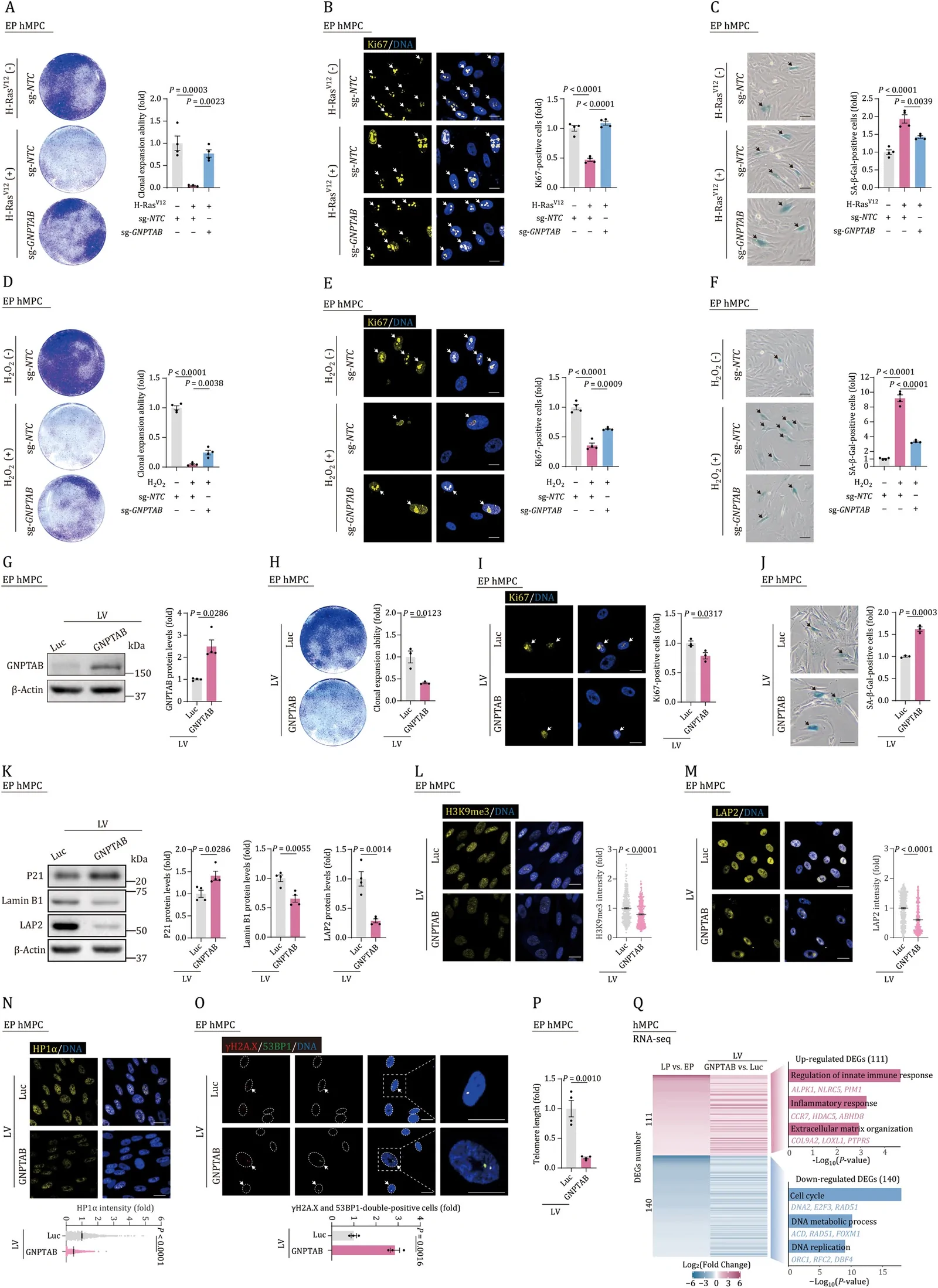
***GNPTAB* depletion mitigates stress-induced senescence, whereas GNPTAB overexpression induces senescence in hMPCs.** (A and D) Clonal expansion analysis of H-Ras^V12^-induced (A) and H_2_O_2_-induced (D) senescence after CRISPRko of *GNPTAB*. (B and E) Immunofluorescence analysis of Ki67 of H-Ras^V12^-induced (B) and H_2_O_2_-induced (E) senescence after CRISPRko of *GNPTAB*. Scale bars, 20 μm. Arrows indicate Ki67-positive cells. (C and F) SA-β-Gal staining of H-Ras^V12^-induced (C) and H_2_O_2_-induced (F) senescence after CRISPRko of *GNPTAB*. Scale bars, 100 μm. Arrows indicate SA-β-Gal-positive cells. (G) Western blot analysis of GNPTAB in EP hMPCs transduced with lentiviruses expressing Luc or GNPTAB. β-Actin was used as the loading control. (H) Clonal expansion analysis in EP hMPCs transduced with lentiviruses expressing Luc or GNPTAB. (I) Immunofluorescence staining of Ki67 in EP hMPCs transduced with lentiviruses expressing Luc or GNPTAB. Scale bars, 20 μm. Arrows indicate Ki67-positive cells. (J) SA-β-Gal staining in EP hMPCs transduced with lentiviruses expressing Luc or GNPTAB. Scale bars, 100 μm. Arrows indicate SA-β-Gal-positive cells. (K) Western blot analysis of P21, Lamin B1, and LAP2 protein levels in EP hMPCs transduced with lentiviruses expressing Luc or GNPTAB. β-Actin was used as the loading control. (L) Immunofluorescence analysis of H3K9me3 in EP hMPCs transduced with lentiviruses expressing Luc or GNPTAB. Scale bars, 20 μm. (M) Immunofluorescence analysis of LAP2 in EP hMPCs transduced with lentiviruses expressing Luc or GNPTAB. Scale bars, 20 μm. (N) Immunofluorescence analysis of HP1α in EP hMPCs transduced with lentiviruses expressing Luc or GNPTAB. Scale bars, 20 μm. (O) Immunofluorescence analysis of γH2A.X and 53BP1 in EP hMPCs transduced with lentiviruses expressing Luc or GNPTAB. Scale bars, 20 μm. Arrows indicate γH2A.X and 53BP1-double-positive foci. (P) qPCR analysis of telomere length in EP hMPCs transduced with lentiviruses expressing Luc or GNPTAB. The expression level was normalized to *RPLP0*. (Q) The heatmap showing the DEGs in GNPTAB-overexpressing hMPCs and RS hMPCs (left). The bar plot on the right displays GO terms and pathways enriched by the shared DEGs between the two groups. The upper and lower panels represent enriched pathways associated with upregulated and downregulated DEGs, respectively. Data in all figures except for (Q) are presented as mean ± SEM. One-way ANOVA followed by Tukey’s multiple-comparison test (A–F), two-tailed Wilcoxon test (G, L–N) and two-tailed unpaired Student’s *t* test (H–K, O, P) were used. *n* = 3–4 biological replicates in (A–K, O, P); *n ≥* 300 cells from three biological replicates in (L–N).

Collectively, these data demonstrate that the geroprotective effect of GNPTAB loss is reproducible and not model-specific. Its ablation mitigates senescence triggered by genetic mutation, oncogenic signaling, oxidative damage, and DNA injury, establishing GNPTAB as a regulator that operates downstream of multiple disparate senescence-inducing pathways in human stem cells.

### GNPTAB overexpression is sufficient to induce senescence

Having established that GNPTAB loss mitigates senescence, we sought to determine its sufficiency in driving senescence. Ectopic expression of GNPTAB in early-passage (EP) hMPCs was sufficient to instigate a comprehensive senescent phenotype (Fig. 2G). This included impaired proliferative capacity (reduced colony formation and Ki67^+^ cells) and an increase in SA-β-Gal activity (Fig. 2H–J). At the molecular level, GNPTAB overexpression upregulated the cell-cycle inhibitor P21 and the classic SASP factors Interleukin 1 beta (*IL1B*) and C-X-C motif chemokine ligand 8 (*CXCL8*), while concurrently depleting heterochromatin markers (H3K9me3, LAP2, HP1α) and nuclear lamina protein Lamin B1 (Figs. 2K–N and S1P). Furthermore, GNPTAB-overexpressing cells displayed heightened genomic instability, with increased DNA damage foci (γH2A.X/53BP1) and accelerated telomere shortening (Fig. 2O and 2P). Global transcriptomic analysis solidified these observations, revealing that GNPTAB overexpression activated inflammatory and senescence-associated genes while suppressing genes involved in cell-cycle progression (Figs. 2Q, S1Q and S1R; Table S4).

Given the canonical role of GNPTAB in lysosomal enzyme trafficking, we investigated whether its pro-senescent effect stemmed from disrupting lysosomal homeostasis. GNPTAB overexpression in young hMPCs induced lysosomal changes, including increased acidity and elevated levels of lysosome-associated membrane protein 2 (LAMP2) and active cathepsin B (CTSB) (Figs. 3A–D and S3A–C) (Kang et al., 2017; Roh et al., 2023; Zhang et al., 2018). However, a catalytically dead mutant (GNPTAB-E937K) that failed to alter these lysosomal parameters (Fig. 3A–D) remained fully capable of inducing senescence (Fig. 3E–H). These results demonstrate that GNPTAB is sufficient to drive cellular senescence, and uncouple its pro-aging function from its classical enzymatic role.

**Figure 3.**
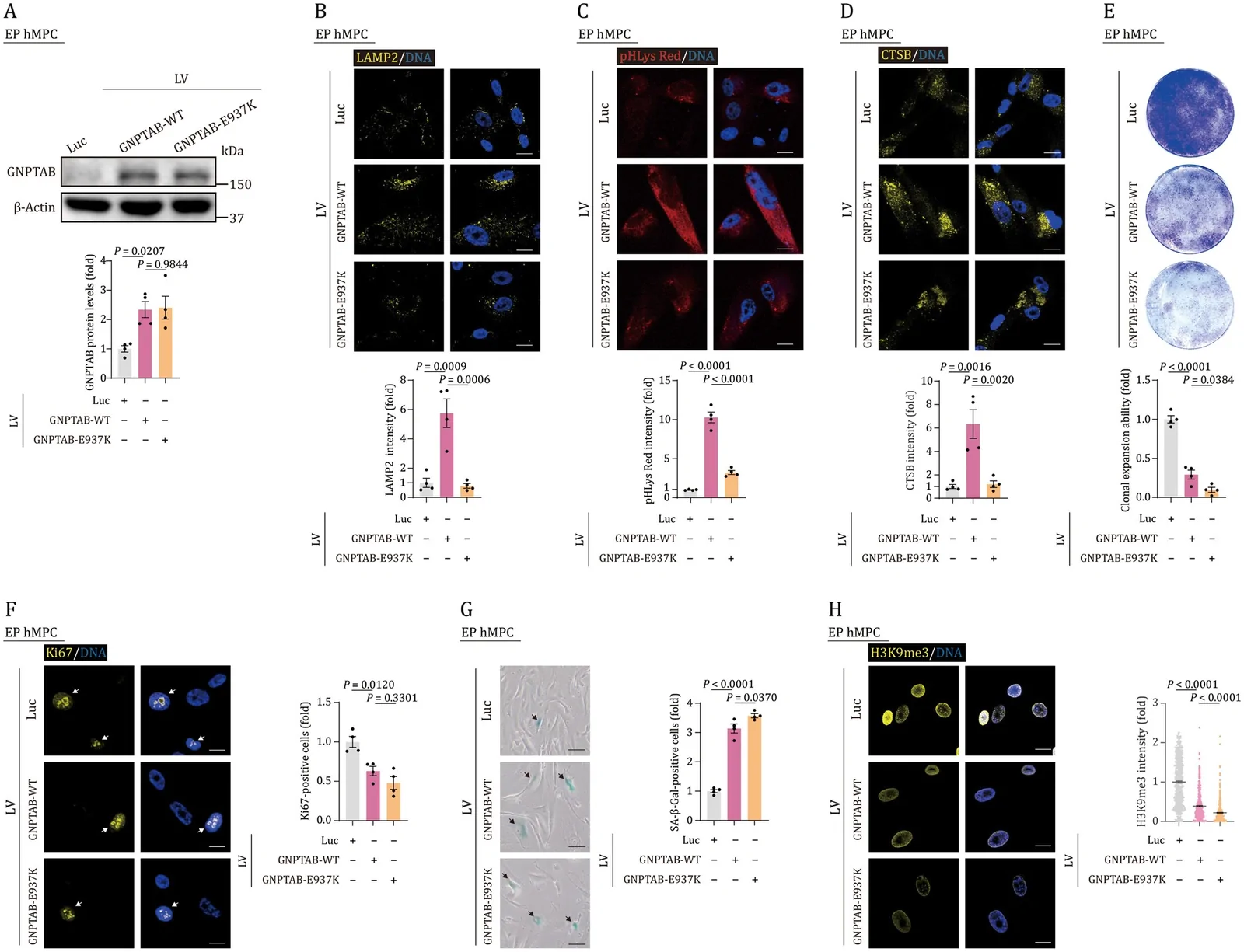
**GNPTAB overexpression accelerates hMPC senescence independent of its canonical catalytic function**. (A) Western blot analysis of GNPTAB in EP hMPCs transduced with lentiviruses expressing Luc, GNPTAB-WT, or GNPTAB-E937K. β-Actin was used as the loading control. (B) Immunofluorescence staining of LAMP2 in EP hMPCs transduced with lentiviruses expressing Luc, GNPTAB-WT, or GNPTAB-E937K. Scale bars, 20 μm. (C) Immunofluorescence staining of pHLys Red in EP hMPCs transduced with lentiviruses expressing Luc, GNPTAB-WT, or GNPTAB-E937K. Scale bars, 20 μm. (D) Immunofluorescence staining of CTSB in EP hMPCs transduced with lentiviruses expressing Luc, GNPTAB-WT, or GNPTAB-E937K. Scale bars, 20 μm. (E) Clonal expansion analysis in EP hMPCs transduced with lentiviruses expressing Luc, GNPTAB-WT, or GNPTAB-E937K. (F) Immunofluorescence staining of Ki67 in EP hMPCs transduced with lentiviruses expressing Luc, GNPTAB-WT, or GNPTAB-E937K. Scale bars, 20 μm. Arrows indicate Ki67-positive cells. (G) SA-β-Gal staining in EP hMPCs transduced with lentiviruses expressing Luc, GNPTAB-WT, or GNPTAB-E937K. Scale bars, 100 μm. Arrows indicate SA-β-Gal-positive cells. (H) Immunofluorescence analysis of H3K9me3 in EP hMPCs transduced with lentiviruses expressing Luc, GNPTAB-WT, or GNPTAB-E937K. Scale bars, 20 μm. Data in all figures are presented as mean ± SEM. One-way ANOVA followed by Tukey’s multiple-comparison test (A–G) and nonparametric Kruskal–Wallis test (H) were used. *n* = 4 biological replicates in (A–G); *n* ≥ 400 cells from four biological replicates in (H).

### GNPTAB drives senescence via an interaction with STING

To elucidate the underlying mechanism of GNPTAB-mediated senescence, we sought to identify its interacting partners. Immunoprecipitation of Flag-tagged GNPTAB followed by mass spectrometry (IP-MS) revealed a proteomic landscape highly enriched for proteins located in the Golgi (Fig. 4A and 4B; Table S5). Notably, among the high-confidence interactors was STING, a central adaptor of cytosolic DNA sensing and inflammatory signaling (Ishikawa and Barber, 2008). The GNPTAB-STING interaction was confirmed by reciprocal co-immunoprecipitation (Co-IP) and immunofluorescence co-localization (Figs. 4C, 4D and S3D).

**Figure 4.**
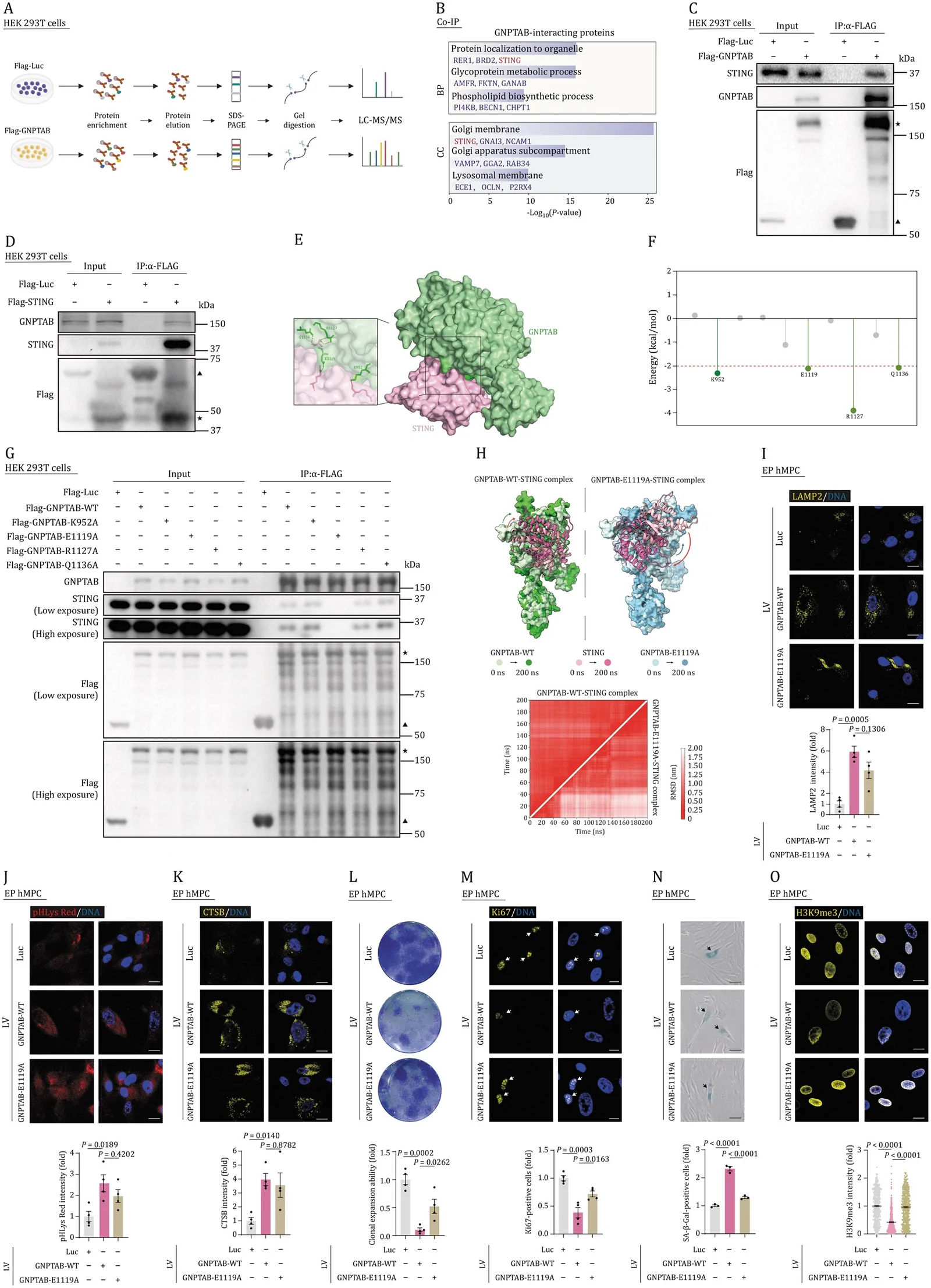
**GNPTAB drives senescence via an interaction with STING**. (A) Schematic diagram of Co-IP assay followed by LC-MS/MS analysis in HEK 293T cells. (B) The bar plot shows the biological process (BP) and cellular components (CC) associated with GNPTAB-interacting proteins identified by Co-IP/MS. (C) Co-IP analysis of the interaction between GNPTAB and STING in HEK 293T cells transduced with Flag-Luc or Flag-GNPTAB. The triangle indicates Flag-Luc, and the asterisk indicates Flag-GNPTAB. (D) Co-IP analysis of the interaction between STING and GNPTAB in HEK 293T cells transduced with Flag-Luc or Flag-STING. The triangle indicates Flag-Luc, and the asterisk indicates Flag-STING. (E) Structural and key residue interaction diagram of the GNPTAB-STING interface. The structural snapshot illustrates the interface between GNPTAB and STING. Key residues are displayed in stick representation, with STING shown in the lower left and GNPTAB shown in the upper right. Dashed lines indicate potential hydrogen-bond interactions. (F) Lollipop plot of contributions of key GNPTAB residues to the binding free energy (Δ*G*) of the complex. The lollipop plot shows the per-residue contributions (kcal/mol) of key GNPTAB residues located at the GNPTAB-STING interface to the overall binding free energy. Positive values indicate unfavorable contributions to binding. (G) Co-IP analysis of the interaction between GNPTAB-WT, GNPTAB-K952A, GNPTAB-E1119A, GNPTAB-R1127A, or GNPTAB-Q1136A and STING in HEK 293T cells transduced with Flag-Luc, Flag-GNPTAB-WT, Flag-GNPTAB-K952A, Flag-GNPTAB-E1119A, Flag-GNPTAB-R1127A, or Flag-GNPTAB-Q1136A. The triangle indicates Flag-Luc, and the asterisk indicates Flag-GNPTAB. (H) Structural snapshots and corresponding time-evolution heat maps of backbone RMSD for the GNPTAB-WT-STING complex and the GNPTAB-E1119A-STING complex. The backbone root-mean-square deviation (RMSD) of GNPTAB-WT and STING proteins over the 200 ns molecular dynamics (MD) simulation (0–200 ns) (left). The right panel depicts the RMSD evolution of the GNPTAB‑E1119A‑STING complex over time. The color bars represent RMSD values (nm), providing a visual representation of conformational stability and the equilibration state of each system. (I) Immunofluorescence staining of LAMP2 in EP hMPCs transduced with lentiviruses expressing Luc, GNPTAB-WT, or GNPTAB-E1119A. Scale bars, 20 μm. (J) Immunofluorescence staining of pHLys Red in EP hMPCs transduced with lentiviruses expressing Luc, GNPTAB-WT, or GNPTAB-E1119A. Scale bars, 20 μm. (K) Immunofluorescence staining of CTSB in EP hMPCs transduced with lentiviruses expressing Luc, GNPTAB-WT, or GNPTAB-E1119A. Scale bars, 20 μm. (L) Clonal expansion analysis in EP hMPCs transduced with lentiviruses expressing Luc, GNPTAB-WT, or GNPTAB-E1119A. (M) Immunofluorescence staining of Ki67 in EP hMPCs transduced with lentiviruses expressing Luc, GNPTAB-WT, or GNPTAB-E1119A. Scale bars, 20 μm. Arrows indicate Ki67-positive cells. (N) SA-β-Gal staining in EP hMPCs transduced with lentiviruses expressing Luc, GNPTAB-WT, or GNPTAB-E1119A. Scale bars, 100 μm. Arrows indicate SA-β-Gal-positive cells. (O) Immunofluorescence staining of H3K9me3 in EP hMPCs transduced with lentiviruses expressing Luc, GNPTAB-WT, or GNPTAB-E1119A. Scale bars, 20 μm. Data in all figures except for (A–H) are presented as mean ± SEM. One-way ANOVA followed by Tukey’s multiple-comparison test (I–N) and nonparametric Kruskal–Wallis test (O) were used. *n* = 3–4 biological replicates in (I–N); *n* ≥ 400 cells from four biological replicates in (O).

To map the binding interface, we employed computational structural modeling. Docking of the GNPTAB and STING crystal structures predicted a plausible complex with four GNPTAB residues potentially contributing to the interface (Fig. 4E). Binding energy decomposition analysis identified four residues (K952, E1119, R1127, Q1136) with the strongest predicted contributions to the interaction (Fig. 4F). Further validation by Co-IP assay revealed that alanine substitution at E1119 (E1119A), but not at other predicted sites, disrupted complex formation with STING (Fig. 4G). Molecular dynamics simulations indicated that the GNPTAB-E1119A mutation induced a potential conformational rearrangement at the binding interface, leading to a less stable complex with elevated backbone fluctuations (Fig. 4H).

Critically, the GNPTAB-E1119A mutant retained the canonical lysosomal enzyme-trafficking function of the wild-type (WT) protein, as evidenced by normal lysosomal acidity (Wang et al., 2019), LAMP2, CTSB, as well as extracellular procathepsin B levels (Figs. 4I–K and S3E). In contrast to GNPTAB-WT, however, expression of GNPTAB-E1119A mutant in EP hMPCs failed to induce senescence, showing no impairment in clonogenicity, proliferation or SA-β-Gal activity, and no loss of heterochromatin marker H3K9me3 (Fig. 4L–O).

These data establish a specific protein-protein interaction between GNPTAB and STING. The functional segregation, where GNPTAB-E1119A loses STING binding and pro-senescent capacity but retains lysosomal function, positions the GNPTAB-STING interface as the essential nexus for its noncanonical, aging-promoting activity.

### The GNPTAB-STING axis activates innate immune and senescence

STING transduces innate immune signals by translocating from the ER to the Golgi apparatus, where it oligomerizes and recruits TBK1 to phosphorylate interferon regulatory factor 3 (IRF3) and nuclear factor kappa B (NF-κB), thereby inducing type I interferon and inflammatory factors expression (Burdette et al., 2011; Civril et al., 2013; Gao et al., 2013; Geng et al., 2025; Guan et al., 2024; Ju et al., 2025; Zhang et al., 2013, 2020). Consistent with the established role of this pathway in aging, senescent hMPCs exhibited heightened phosphorylation of STING, TBK1, IRF3, and NF-κB, along with elevated expression of downstream interferon-stimulated genes (ISGs) and other type I interferon genes (Fig. S3F–K) (Wang et al., 2025).

We therefore asked whether GNPTAB triggers senescence by activating this STING-dependent inflammatory cascade. Indeed, ectopic GNPTAB-WT expression in young hMPCs activated the STING signaling, marked by increased phosphorylation of STING, TBK1, IRF3, and NF-κB, together with the induction of ISGs (Interferon-induced protein with tetratricopeptide repeats 1 (*IFIT1*), Interferon-stimulated gene 15 (*ISG15*)) and pro-inflammatory cytokines (Interferon alpha 1 (*IFNA1*), Interferon beta 1 (*IFNB1*), Tumor necrosis factor (*TNF*)) (Fig. 5A and 5B). Importantly, the STING-binding-deficient mutant GNPTAB-E1119A was inert in this regard, confirming that signaling activation requires their interaction (Fig. 5A and 5B). Conversely, genetic ablation of GNPTAB in senescent hMPCs suppressed the phosphorylation of STING and downstream components and downregulated the expression of inflammatory factors (Fig. S3L and S3M). Notably, GNPTAB overexpression did not alter intracellular levels of 2′3′-cyclic guanosine monophosphate-adenosine monophosphate (cGAMP) (Fig. S3N), suggesting that the canonical upstream activation of STING may not be engaged under conditions of elevated GNPTAB expression. Consistent with this possibility, siRNA-mediated knockdown of cGAS (encoded by *MB21D1*) in GNPTAB-overexpressing cells had no detectable effect on STING phosphorylation (Fig. 5C and 5D). Likewise, GNPTAB-induced cellular senescence was unaffected by cGAS depletion (Fig. 5E). These findings indicate that GNPTAB activates STING in a cGAS-independent way. Immunofluorescence analysis further revealed that GNPTAB overexpression promoted STING localization to the Golgi apparatus, where it partially colocalized with GNPTAB (Fig. 5F), suggesting that GNPTAB may facilitate STING activation by promoting its Golgi translocation.

**Figure 5.**
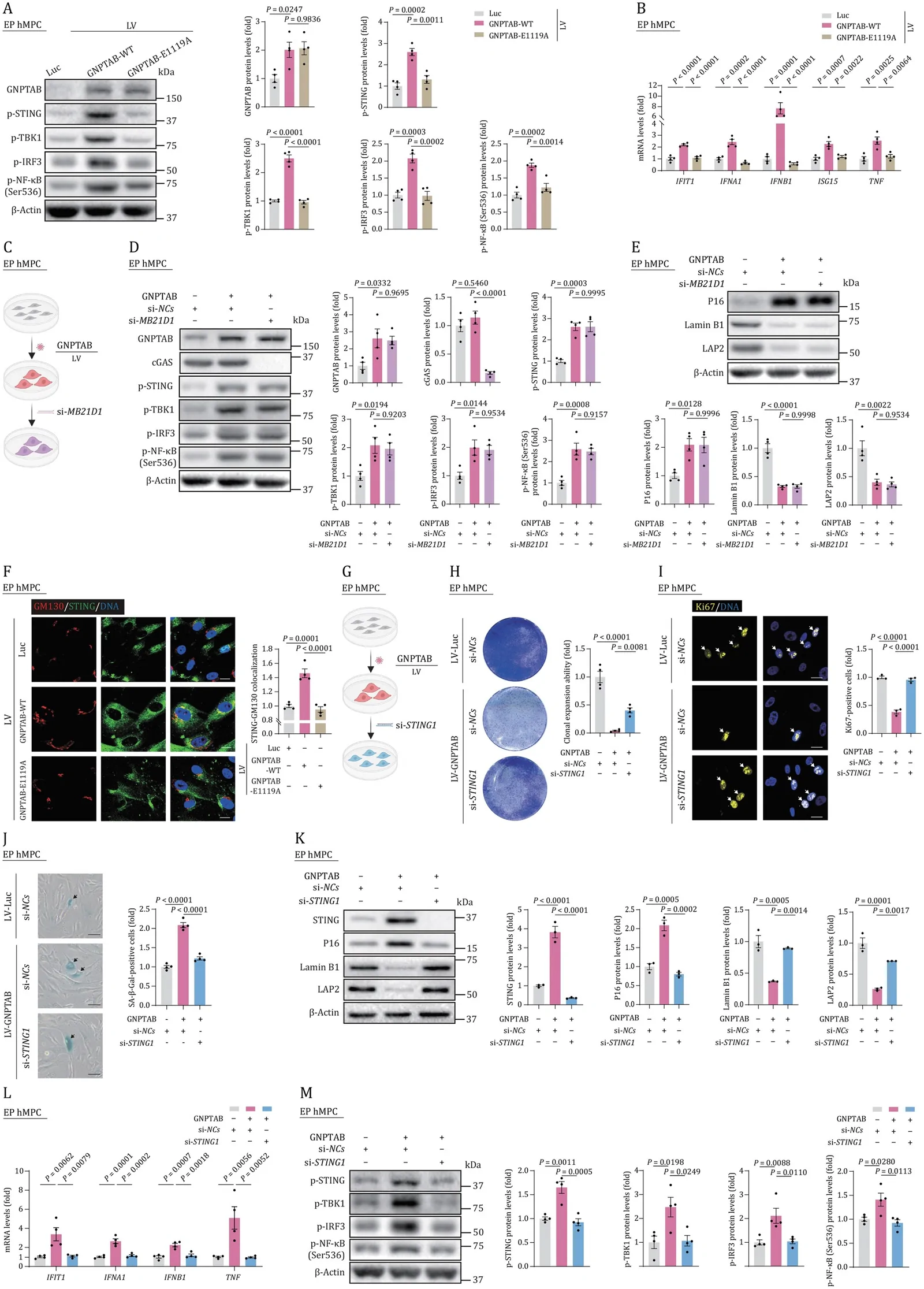
**The GNPTAB-STING axis activates innate immune signaling and promotes senescence**. (A) Western blot analysis of GNPTAB, p-STING, p-TBK1, p-IRF3 and p-NF-κB protein levels in EP hMPCs transduced with lentiviruses expressing Luc, GNPTAB-WT or GNPTAB-E1119A. β-Actin was used as the loading control. (B) Relative mRNA levels of *IFIT1*, *IFNA1*, *IFNB1, ISG15* and *TNF* in EP hMPCs transduced with lentiviruses expressing Luc, GNPTAB-WT or GNPTAB-E1119A. (C) Schematic of *MB21D1* suppression with siRNAs in GNPTAB-transduced EP hMPCs. (D) Western blot analysis of GNPTAB, cGAS, p-STING, p-TBK1, p-IRF3 and p-NF-κB protein levels of GNPTAB-transduced EP hMPCs transfected with si-*MB21D1*. β-Actin was used as the loading control. (E) Western blot analysis of the P16, Lamin B1 and LAP2 protein levels of GNPTAB-transduced EP hMPCs transfected with si-*MB21D1*. β-Actin served as the loading control. (F) Immunofluorescence staining of golgi matrix protein 130 (GM130) and STING in EP hMPCs transduced with lentiviruses expressing Luc, GNPTAB-WT or GNPTAB-E1119A. Scale bars, 20 μm. (G) Schematic of knockdown of *STING1* with siRNAs in GNPTAB-transduced EP hMPCs. (H) Clonal expansion analysis of GNPTAB-transduced EP hMPCs transfected with si-*STING1*. (I) Immunofluorescence staining of Ki67 in GNPTAB-transduced EP hMPCs transfected with si-*STING1*. Scale bars, 20 μm. Arrows indicate Ki67-positive cells. (J) SA-β-Gal staining of GNPTAB-transduced EP hMPCs transfected with si-*STING1*. Scale bars, 100 μm. Arrows indicate SA-β-Gal-positive cells. (K) Western blot analysis of STING, P16, Lamin B1 and LAP2 protein levels in GNPTAB-transduced EP hMPCs transfected with si-*STING1*. β-Actin was used as the loading control. (L) Relative mRNA levels of *IFIT1*, *IFNA1*, *IFNB1* and *TNF* in GNPTAB-transduced EP hMPCs transfected with si-*STING1*. (M) Western blot analysis of p-STING, p-TBK1, p-IRF3 and p-NF-κB protein levels of GNPTAB-transduced EP hMPCs transfected with si-*STING1*. β-Actin was used as the loading control. Data in all figures except for (C and G) are presented as mean ± SEM. One-way ANOVA followed by Tukey’s multiple-comparison test (A, B, D–F, H–M) was used. *n* = 3–4 biological replicates in (A, B, D–F, H–M).

To establish the functional requirement for STING, we performed loss-of-function epistasis analysis. Knockdown of STING (encoded by *STING1*) in GNPTAB-overexpressing cells abolished the pro-senescent effects, restoring clonogenic growth, proliferation, and re-establishing nuclear lamina integrity (Lamin B1, LAP2), while reducing SA-β-Gal activity and P16 expression (Fig. 5G–K). Concomitantly, STING inhibition abrogated the activation of its downstream phosphorylation cascade and suppressed the induction of inflammatory genes (Fig. 5L and 5M).

Together, these complementary gain- and loss-of-function experiments converge on a unified mechanistic model in which GNPTAB engages STING, leading to its activation and the initiation of the inflammatory signaling that executes cellular senescence (Fig. S3O). These findings uncover a noncanonical mechanism of STING activation that links the organellar trafficking factor to chronic innate immune signaling in cellular aging.

## Discussion

This study uncovers the aging-promoting function of the lysosomal enzyme-targeting factor GNPTAB. Through a focused CRISPR screen for lysosome-associated genes, we demonstrate that GNPTAB is a driver of cellular senescence. Crucially, we dissect two separable functions of this protein: its well-established role in lysosomal hydrolase trafficking and a novel signaling function mediated by binding and activation of the innate immune adaptor STING (Li et al., 2022; Lv et al., 2026; Zhang et al., 2025; Zhang et al., 2025). The identification of a STING-binding-deficient mutant (GNPTAB-E1119A) that is catalytically competent yet senescence-inert establishes the GNPTAB-STING axis as a central mechanism linking a lysosomal machinery component to inflammatory aging.

Our findings expand the functional repertoire of lysosome-associated proteins. While traditionally viewed through the lens of degradation and nutrient sensing (Lawrence and Zoncu, 2019; Maejima et al., 2013; Parenti et al., 2015; Settembre et al., 2013), our work positions GNPTAB as a bona fide signaling node that directly engages a major innate immune pathway. This reveals a parallel route of STING activation distinct from the canonical cytosolic DNA-cGAS signaling (Zhang et al., 2020; Zhang et al., 2023), suggesting that organelle-associated factors may serve as critical upstream rheostats for innate immune signaling in aging contexts. Golgi apparatus, as a key intracellular organelle, may function as an important signaling platform in multiple cellular processes. In particular, Golgi-resident proteins such as GNPTAB may regulate senescence-associated inflammatory signaling by interacting with the critical node of inflammatory pathways.

Beyond GNPTAB, our screening platform yielded additional candidates, such as *HYAL3*, that may orchestrate similar organelle-immune crosstalk (Rahmioglu et al., 2023; Romo et al., 2022), highlighting a potentially broader regulatory network. The physiological relevance of the GNPTAB-STING requires *in vivo* validation. Studies in aging murine models or age-related disease contexts (e.g., osteoarthritis, fibrosis) will determine whether modulating this interaction can ameliorate tissue dysfunction driven by cellular senescence (Cai et al., 2023; Diao et al., 2021; Gulen et al., 2023; Hu et al., 2020; Jing et al., 2023; Liang et al., 2021; Wang et al., 2021). The functional segregation of GNPTAB’s enzymatic activity and pro-inflammatory signaling is particularly encouraging from a therapeutic perspective, as it raises the possibility of developing targeted inhibitors that disrupt the pro-senescent interaction while sparing essential lysosomal function (Wang and Du, 2025).

In summary, this work redefines GNPTAB from a lysosomal protein-modifying enzyme to an active signaling node that directly licenses STING-dependent senescence, revealing a previously unrecognized immune regulatory node with broad potential for mitigating age-related decline.

## Supplementary Material

pwag049_Supplementary_Data

## Data Availability

CRISPR-based screening and RNA-seq data have been submitted to the Genome Sequence Archive in the National Genomics Data Center, Beijing Institute of Genomics (China National Center for Bioinformation) of the Chinese Academy of Sciences, with accession number HRA016009. The mass spectrometry proteomics data have been deposited to the ProteomeXchange Consortium via the iProX partner repository with the dataset identifier PXD073027.

## Code availability

All the custom codes are available from the corresponding authors upon reasonable request.

## Abbreviations

ANOVA: analysis of variance;
BP: biological process;
cGAMP: cyclic guanosine monophosphate-adenosine monophosphate;
cGAS: cyclic GMP-AMP synthase;
CC: cellular component;
Co-IP: co-immunoprecipitation;
CRISPR: clustered regularly interspaced short palindromic repeats;
CRISPRko: CRISPR-mediated knockout;
CTSB: Cathepsin B;
*CTSL*: Cathepsin L;
*CXCL8*: C-X-C motif chemokine ligand 8;
ELISA: enzyme-linked immunosorbent assay;
EP: early-passage;
gDNA: genomic DNA;
GM130: Golgi matrix protein 130;
GNPTAB: N-acetylglucosamine-1-phosphotransferase subunits alpha and beta;
GO: gene ontology; γ
H2A.X: phosphorylated histone H2A.X; H_2_O_2_, hydrogen peroxide;
H3K9me3: trimethylation of histone H3 lysine 9;
hESCs: human embryonic stem cells;
hMPCs: human mesenchymal progenitor cells;
HGPS: Hutchinson-Gilford progeria syndrome;
HP1α: heterochromatin protein 1 alpha;
*HYAL3*: Hyaluronidase 3;
*IFIT1*: Interferon-induced protein with tetratricopeptide repeats 1;
*IFNA1*: Interferon alpha 1;
*IFNB1*: Interferon beta 1;
*IL1B*: Interleukin 1 beta;
IP-MS: immunoprecipitation-mass spectrometry;
IRF3: interferon regulatory factor 3;
*ISG15*: Interferon-stimulated gene 15;
ISGs: interferon-stimulated genes;
KEGG: Kyoto Encyclopedia of Genes and Genomes;
LAGs: lysosome-associated genes;
LAMP2: lysosome-associated membrane protein 2;
LAP2: lamina-associated polypeptide 2;
*LMNA*: lamin A/C;
LOF: loss-of-function;
LP: late-passage;
M6P: mannose-6-phosphate;
MOI: multiplicity of infection;
*NAGPA*: N-acetylglucosamine-1-phosphodiester alpha-N-acetylglucosaminidase;
NF-κB: nuclear factor kappa B;
NGS: next-generation sequencing;
NTC: non-targeting control;
RRA: robust rank aggregation;
RS: replicative senescence;
RT-qPCR: real-time quantitative polymerase chain reaction;
SA-β-Gal: senescence-associated β-galactosidase;
SASP: senescence-associated secretory phenotype;
SDS-PAGE: sodium dodecyl sulfate polyacrylamide gel electrophoresis;
sgRNA: single guide RNA;
*SLC38A9*: solute carrier family 38 member 9;
STING: stimulator of interferon genes;
TBK1: TANK-binding kinase 1;
TNF: Tumor necrosis factor;
UV: ultraviolet;
53BP1: tumor protein p53-binding protein 1; WT, wild-type.
